# Tail Artifact Removal via Transmittance Effect Subtraction in Optical Coherence Tail Artifact Images

**DOI:** 10.3390/s23239312

**Published:** 2023-11-21

**Authors:** Urban Simoncic, Matija Milanic

**Affiliations:** 1 Faculty of Mathematics and Physics, University of Ljubljana, 1000 Ljubljana, Slovenia; matija.milanic@fmf.uni-lj.si; 2 Jozef Stefan Institute, 1000 Ljubljana, Slovenia

**Keywords:** Optical Coherence Tomography (OCT), Optical Coherence Tomography Angiography (OCTA), Tail Artifact Removal, physics-based image processing

## Abstract

Optical Coherence Tomography Angiography (OCTA) has revolutionized non-invasive, high-resolution imaging of blood vessels. However, the challenge of tail artifacts in OCTA images persists. In response, we present the Tail Artifact Removal via Transmittance Effect Subtraction (TAR-TES) algorithm that effectively mitigates these artifacts. Through a simple physics-based model, the TAR-TES accounts for variations in transmittance within the shallow layers with the vasculature, resulting in the removal of tail artifacts in deeper layers after the vessel. Comparative evaluations with alternative correction methods demonstrate that TAR-TES excels in eliminating these artifacts while preserving the essential integrity of vasculature images. Crucially, the success of the TAR-TES is closely linked to the precise adjustment of a weight constant, underlining the significance of individual dataset parameter optimization. In conclusion, TAR-TES emerges as a powerful tool for enhancing OCTA image quality and reliability in both clinical and research settings, promising to reshape the way we visualize and analyze intricate vascular networks within biological tissues. Further validation across diverse datasets is essential to unlock the full potential of this physics-based solution.

## 1. Introduction

Optical Coherence Tomography (OCT) has emerged as a non-invasive imaging tool, offering high-resolution views of biological tissues and materials [1,2]. Operating on low-coherence interferometry, OCT provides us with precise cross-sectional images of internal structures.

OCT imaging goes beyond static imaging by detecting motion within samples, enabling real-time imaging of dynamic processes. One such OCT technique is Optical Coherence Tomography Angiography (OCTA), a potent technique for visualizing blood vessels and microvascular networks in living tissues [3,4,5,6]. It relies on dynamic contrast, where the movement of red blood cells (RBCs) in vessels causes fluctuations in OCT signals, distinguishing blood vessels from surrounding tissues [5,7,8,9]. OCTA is widely used in ophthalmology, neuroscience, cancer research, and dermatology [8,9,10,11,12].

OCTA faces a significant challenge—occurrence of imaging artifacts known as projection artifacts or tail artifacts. These artifacts occur for various reasons, including variation of transmitted incident light to deeper layers due to the variable transmittance in the shallow layers with the vasculature [13,14] and elongated light paths from multiple scattering of photons interacting with flowing RBCs [15,16]. Variation of transmitted incident light to deeper layers due to the variable transmittance in the shallow layers causes OCT signal variation even in the absence of reflectivity variation, generating vasculature-like signals beneath the vasculature. Elongated light paths from multiple scattering of photons increase the delay of reflected light from the vessels in shallow layers and attribute that signal to deep layers.

Several strategies exist to mitigate those imaging artifacts and often involve image processing techniques and hardware solutions. An example of a hardware solution is a high numerical aperture objective that minimizes multiple scattering effects. However, the high numerical aperture imaging also requires dynamic focusing to reconstruct the OCTA, which may not always be a trivial task and therefore limits widespread adoption [17]. In addition, it only removes the effects of elongated light paths from multiple scattering of photons. Early examples of image processing techniques are Step-down Exponential filtering [18], slab-subtraction [19,20], and projection-resolved algorithm [21]. The slab-subtraction and projection-resolved algorithm are quite popular, especially in ophthalmological imaging. Our experiences with these algorithms on skin OCTA images are less promising. The Step-down Exponential filtering is also partially effective but may cause signal loss, especially in deep vascular regions. A more recent Mean-Subtraction method removes tail artifacts by subtracting the weighted mean value of A-scan but may disrupt deep flows and hinder quantitative blood flow analysis [16]. Deep learning-based OCTA can suppress tail artifacts effectively but demands computational resources and extensive annotated datasets, making manual labeling labor intensive and prone to errors [22].

In this work, we introduce an innovative physics-based OCTA tail artifact removal algorithm. The algorithm relies on a simple OCTA physical model for the main source of the tail artifact—the variation of transmitted incident light to deeper layers due to the variable transmittance in the shallow layers with the vasculature. We demonstrate our algorithm’s effectiveness on hemangioma vasculature OCTA images. As a validated and simple solution backed with a physical model, this algorithm provides a new solution for tail artifact suppression and improved blood vessel network visualization.

## 2. Materials and Methods

### 2.1. Tail Artifact Removal via Transmittance Effect Subtraction (TAR-TES) Algorithm

The new OCTA tail removal algorithm, the Tail Artifact Removal via Transmittance Effect Subtraction (TAR-TES), calculates the corrected OCTA image by subtracting the contribution of transmittance variation from the OCTA image. The structural image intensity (also known as OCT image intensity) in the *n*-th layer, *S_n_*, is proportional to the fraction of incident light transmitted to the *n*-th layer, *T_n_*, and the reflectivity of the *n*-th layer, *R_n_*, with $w_{1}^{-1}$ being the proportionality factor. We write proportionality factor as the inverse of *w*_1_ to ensure that the final equation has it as a linearly proportional factor.

(1) $$S_{n}=\frac{T_{n}\cdot R_{n}}{w_{1}}$$

The angiographic image intensity (also known as OCTA image intensity) in the *n*-th layer, *A_n_*, is proportional to the variation in the structural image.

(2) $$A_{n}=\frac{\delta \left(T_{n}\cdot R_{n}\right)}{w_{1}}=\frac{\delta T_{n}\cdot R_{n}+T_{n}\cdot \delta R_{n}}{w_{1}}$$

We assume that the corrected OCTA image intensity is proportional to the variation in reflectivity. From this, we can calculate the corrected OCTA by subtracting the variable transmittance effect from the uncorrected angiographic image.

(3) $$A_{n}^{corr}=\frac{T_{n}\cdot \delta R_{n}}{w_{1}}=A_{n}-\frac{\delta T_{n}\cdot R_{n}}{w_{1}}$$

The variation in the transmitted light to the *n*-th layer *δT_n_* depends on the variation in reflected light in previous layers, i.e., on the corrected angiographic image. It is calculated as the square root of the sum of squared variations in reflectivity from layers 1 to *n* − 1, assuming that the variations in different layers are uncorrelated.

(4) $$\delta T_{n}=\delta \left(1-\sum_{i=1}^{n-1}R_{i}\right)=\sqrt{\sum_{i=1}^{n-1}\delta R_{i}^{2}}=w_{1}\cdot \sqrt{\sum_{i=1}^{n-1}\frac{{\left(A_{i}^{corr}\right)}^{2}}{T_{i}^{2}}}$$

We can insert Equations (1) and (4) into Equation (3) to substitute the variation in the transmitted light to the *n*-th layer *δT_n_* with the corrected OCTA from the previous layers and the reflectivity *R_n_* with the structural image *S_n_*. Thus, we obtain the formula for the correction of the *n*-th layer in the OCTA image.

(5) $$A_{n}^{corr}=A_{n}-w_{1}\cdot \frac{S_{n}}{T_{n}}\cdot \sqrt{\sum_{i=1}^{n-1}\frac{{\left(A_{i}^{corr}\right)}^{2}}{T_{i}^{2}}}$$

We further assume that almost all incident light is transmitted light to the *n*-th layer, so we set $T_{i}=1$. In that way, we obtain the simplified formula for the corrected OCTA image. It is calculated by subtracting scaled structural image *S_n_*, multiplied by the square root of the sum of squared corrected angiographic images from layers 1 to *n* − 1 from *A_n_*.

(6) $$A_{n}^{corr}=A_{n}-w_{1}\cdot S_{n}\cdot \sqrt{\sum_{i=1}^{n-1}{\left(A_{i}^{corr}\right)}^{2}}$$

This new algorithm provides a novel approach for removing tail artifacts in OCTA images by accounting for the contribution of variable transmittance to the deep layers in OCTA images. It has one free parameter, *w*_1_, which is adjusted to control the strength of the OCTA tail-removal correction.

### 2.2. Vasculature Model

In this study, we imaged the hand’s birthmark, namely hemangioma, a vascular anomaly characterized by an abnormal overgrowth of blood vessels. Hemangiomas can generally occur in various tissues, including the skin, and are often characterized by a complex network of blood vessels. Hemangiomas are generally harmless, and they offer a good model for blood vasculature [23]. OCTA offers a non-invasive means of imaging hemangiomas, providing high-resolution, depth-resolved images of the vasculature.

**Figure 1:**
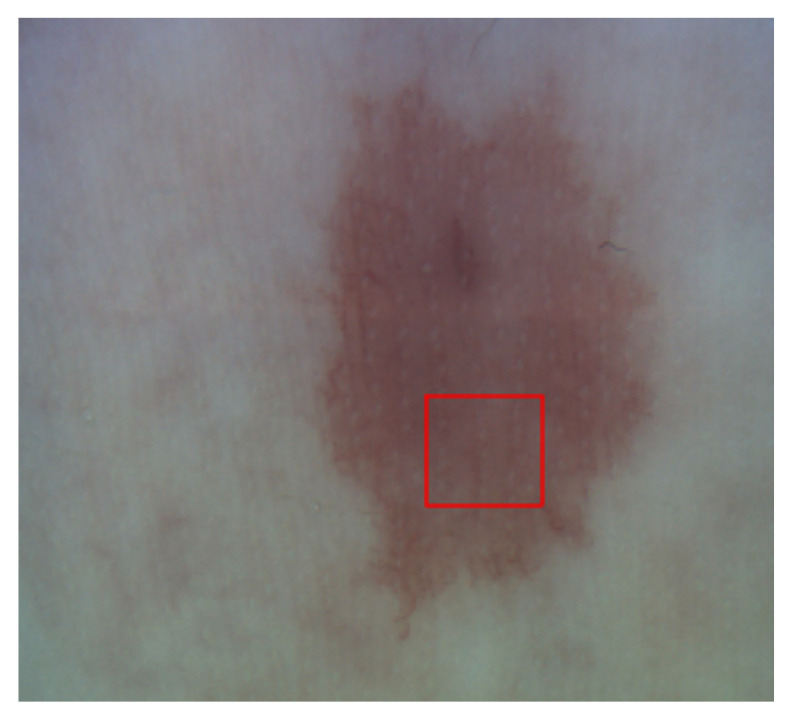
Picture of hemangioma that was imaged in this study. The red box is marking the OCT field of view.

The particular hemangioma imaged in this study (Figure 1) served as a representative sample for assessing the efficacy of the Tail Artifact Removal via Transmittance Effect Subtraction (TAR-TES) algorithm in correcting tail artifacts in OCTA images. The complex and intricate vascular structure within the hemangioma allowed for a comprehensive evaluation of the algorithm’s performance in a real-world clinical context. Although analysis of only one vascular image is presented here to demonstrate the performance of the TAR-TES algorithm, similar results were obtained for other vascular lesion OCTA images.

### 2.3. Speckle-Variance OCT Angiography

Optical Coherence Tomography Angiography (OCTA) was used in this study. The imaging was conducted using a commercial Thorlabs VEG220C1 swept-source OCT system, which featured a central wavelength of 1300 nm. The selection of this particular wavelength offers distinct advantages, notably enhanced tissue penetration and improved contrast for visualizing vasculature structures, making it well-suited for in-depth vascular imaging.

To capture angiographic information, a speckle variance OCTA technique was utilized. The OCTA imaging was acquired with a series of five image repetitions. The image was reconstructed with the software that was provided with the OCT system (ThorImage OCT Version 5.5.5.0). The imaging voxel size was set at 2.3 μm × 2.2 μm × 8.1 μm, and the image size was 357 × 373 × 960. OCT and OCTA images were filtered with a Gaussian filter with sigma equals one voxel size. The noise level was reduced by subtracting the average imaging signal deep inside the tissue (depth 4–7 mm), where only homogeneous noise is present in the image.

### 2.4. Tail Artifact Correction in OCTA Image

Tail artifact correction via the TAR-TES algorithm was performed by applying Equation (5) for each consecutive layer. Weight *w*_1_ was adjusted manually; it was increased until tail artifacts visually disappeared (default *w*_1_ value). We also tested 2-times lower *w*_1_ value and 2.5-times higher *w*_1_ value.

The TAR-TES algorithm was compared to two most similar competitive algorithms: the Mean-Subtraction algorithm [16] and the Step-down Exponential filtering method [18]. They were implemented using Equations (6) and (7). One free parameter in those algorithms, *w*_2_ or *w*_3_, was adjusted manually to achieve roughly the same level of signal decrease as with the default *w*_1_ value.

In comparison to the TAR-TES algorithm, the Mean-Subtraction algorithm reduces tail artifacts in OCTA images by subtracting the weighted mean of the depth profile.

(7) $$A^{corr}=A-\frac{w_{2}}{N}\sum_{i=1}^{N}A_{i}$$

Like the TAR-TES algorithm, it has one free parameter, *w*_2_, which is adjusted to control the strength of the OCTA tail-removal correction. Unlike the TER-TES algorithm, the Mean-Subtraction algorithm reduces tail artifacts by subtracting the same value, the mean of the uncorrected depth profile, from all layers. In contrast, the TAR-TES algorithm subtracts the contribution of transmittance variation to the angiographic image separately for each consecutive layer and uses corrected images from previous layers.

An older Step-down Exponential filtering method, which is another well-established method for OCTA tail removal, attenuates the OCTA signal of each consecutive layer by a factor proportional to the exponential sum of values from the corrected images from previous layers.

(8) $$A_{n}^{corr}=A_{n}\times e^{-w_{3}\times \sum_{i=1}^{n-1}A_{i}^{corr}}$$

Like the TAR-TES algorithm, the Step-down Exponential filtering method performs correction for each consecutive layer and uses corrected images from previous layers. Similarly, it has one free parameter, *w*_3_, which is adjusted to control the strength of the OCTA tail-removal correction. In contrast to the TAR-TES algorithm, the Step-down Exponential filtering method performs correction by scaling each layer, not by subtraction.

## 3. Results

**Figure 2:**
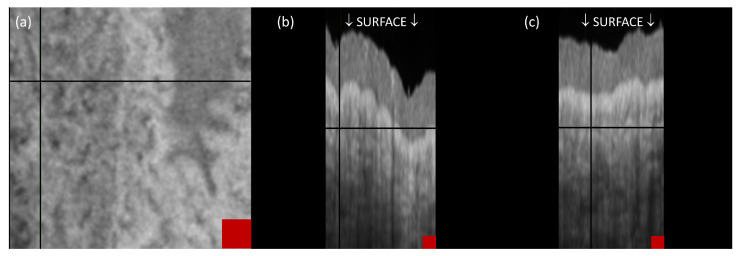
Structure OCT images: en face (**a**), B-scan cross-sectional (**b**) and C-scan cross-sectional (**c**) view. Crosses on each view represent the location of the section for the other two views. All three sections define one point, which is in the vessel that is not visible in the OCT image. The red squares represent an area of 100 μm × 100 µm.

The structural OCT image *S* of the hemangioma in all three views is shown in Figure 2. Each view is marked with a cross symbolizing the position of the section corresponding to the other two views. These three sections collectively define a single point within the vessel that is not discernible in the OCT image.

**Figure 3:**
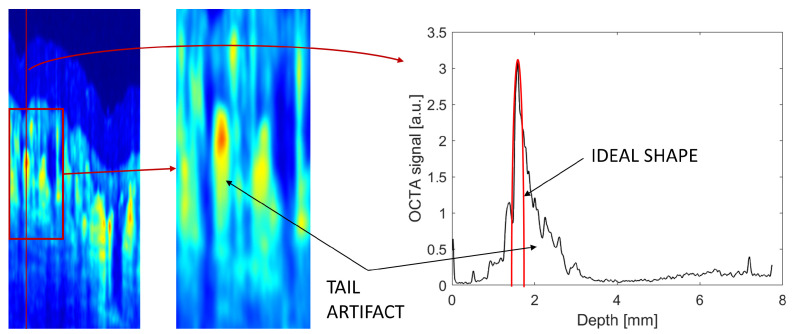
The OCTA tail artifact is visible as a slowly decreasing signal after the large vessel. The OCTA depth profile exhibits a sharp increase at the position of the vessel and a slow decrease in the signal beneath the vessel. The ideal profile would be a symmetric peak in place of the vessel (sketched in red on the depth profile).

The uncorrected OCTA image exhibits substantial tails beneath the larger superficial vessels and lingering shadow artifacts in deep layers. This is a known OCTA artifact, and a proper vasculature image would show a sharp decrease in the OCTA signal after the vessels—as we see a sharp increase in the signal at the front edge of the vessel. A schematic representation of the OCTA tail is shown in Figure 3.

**Figure 4:**
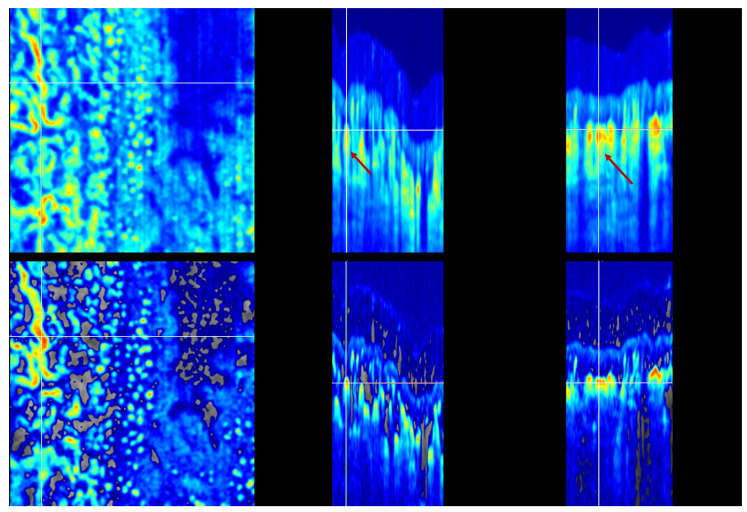
Uncorrected and corrected OCTA images: en face (**left**), B-scan cross-sectional (**middle**) and C-scan cross-sectional (**right**) view. The upper row shows uncorrected OCTA with one example of the tail artifact marked with an arrows. The lower row shows corrected OCTA images with the TAR-TES method and *w*_1_ = 0.02. The removal of tail artifacts is balanced. (Note that the color scale is different for each row).

**Figure 5:**
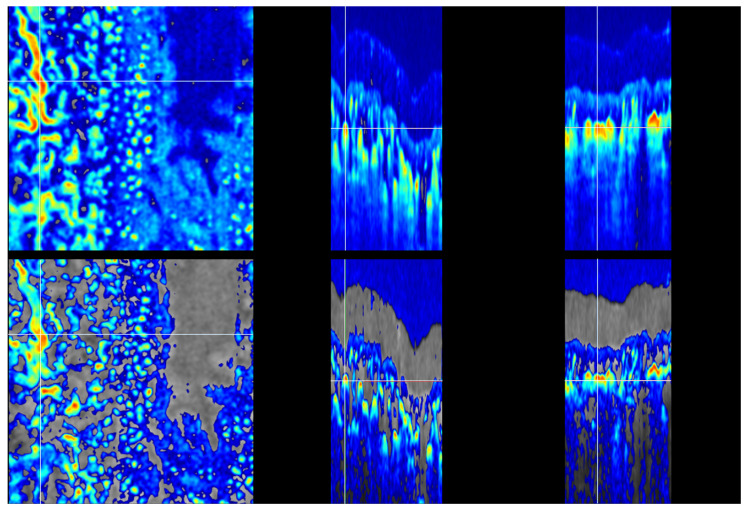
Corrected OCTA images with the TAR-TES method and *w*_1_ = 0.01 (**upper row**), and *w*_1_ = 0.05 (**lower row**). On the left is the en face view, in the middle is the B-scan cross-sectional view, and on the right is the C-scan cross-sectional view. (Note that the color scale is different for each row).

The TAR-TES algorithm performance for tail artifact removal was examined with weight constants (*w*_1_) set at 0.01, 0.02, and 0.05 (Figure 4 and Figure 5). The correction with an adequately adjusted weight constant for our proposed TAR-TES algorithm must effectively suppress tail artifacts while keeping the vasculature image.

The removal of tail artifacts for weight *w*_1_ = 0.02 is shown in Figure 4. The vessel marked with the cross is clearly visible on the en face view, and the B-scan cross-sectional and C-scan cross-sectional views show the expected shape of the vessel—a shape that resembles the circle. Tail artifacts are slightly visible but far less than on the OCTA with no correction.

The removal of tail artifacts for different weight conditions (*w*_1_ = 0.01 and *w*_1_ = 0.05) is shown in Figure 5. At the weight value of *w*_1_ = 0.01, we observe partial tail artifact removal, indicating that the weight is insufficient. At the weight value of *w*_1_ = 0.05, we observe complete tail artifact removal and missing OCTA signal in some parts of the image, which indicates that the image is over-corrected. Also, the cross-section of the vessel is a flattened circle, which is another indicator that the weight might be too high.

**Figure 6:**
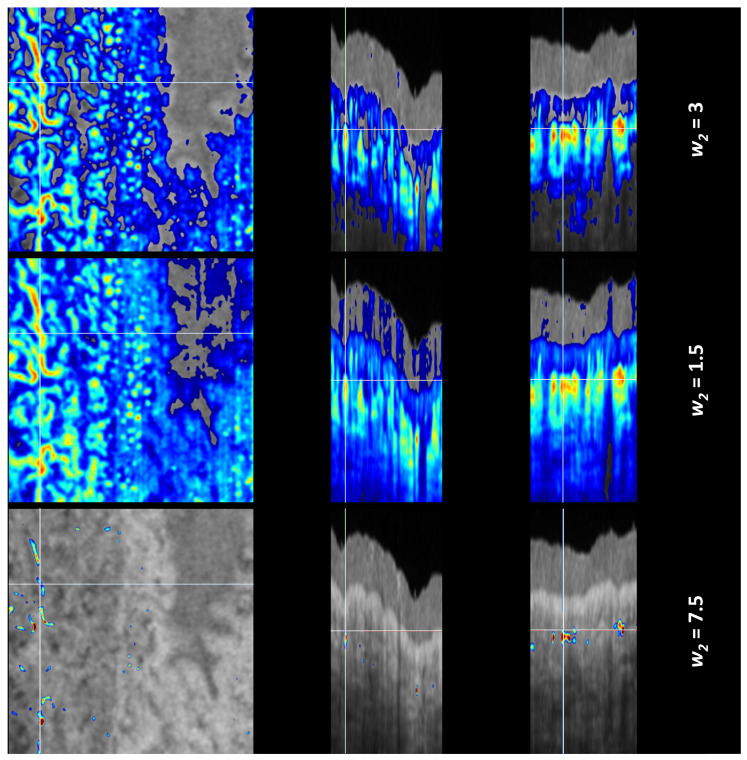
Corrected OCTA images with the Mean-Subtraction algorithm for three correction weights. On the left is the en face view, in the middle is the B-scan cross-sectional view, and on the right is the C-scan cross-sectional view. The first row shows the corrected OCTA with the weight that we consider optimal, the second row shows the correction with two-times lower weight, and the third row shows the correction with a 2.5-times higher weight. (Note that the color scale is different for each row).

**Figure 7:**
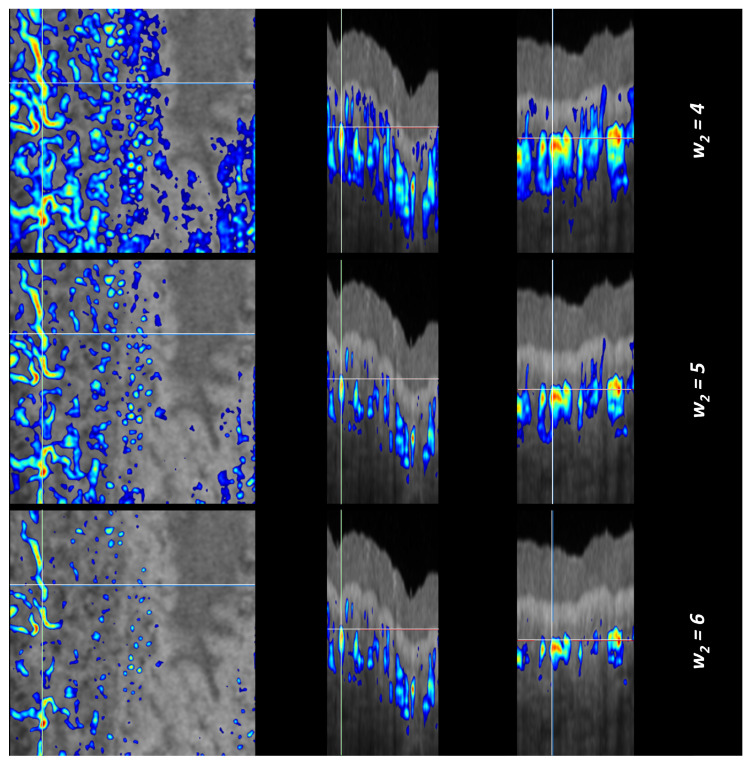
Corrected OCTA images with the Mean-Subtraction algorithm for three additional correction weights. (Note that the color scale is different for each row).

**Figure 8:**
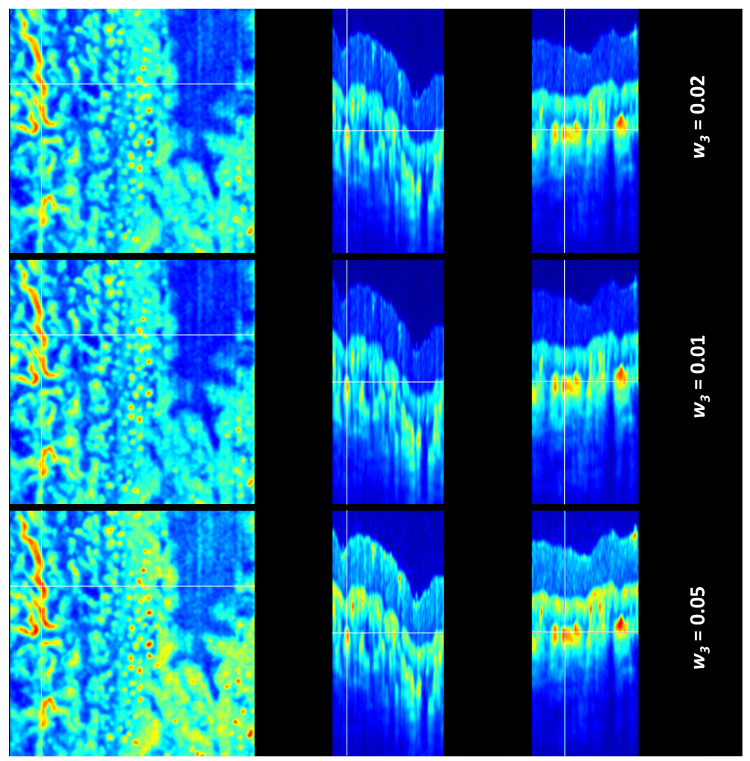
Corrected OCTA images with the Step-down Exponential filtering method. On the left is the en face view, in the middle is the B-scan cross-sectional view, and on the right is the C-scan cross-sectional view. The first row shows the corrected OCTA with the weight that we consider optimal, the second row shows the correction with two-times lower weight, and the third row shows the correction with a 2.5-times higher weight. (Note that the color scale is different for each row).

To evaluate the TAR-TES algorithm performance, we compared it to the Mean-Subtraction algorithm [16] and the Step-down Exponential filtering method [18]. Weights for both correction methods were adjusted manually; they were increased until the tail artifacts visually disappeared. As with the TAR-TES algorithm, we also did the correction with 2-times smaller weight and with the 2.5-times larger weight. The results are shown in Figure 6, Figure 7 and Figure 8. Due to the big difference in results for the Mean-Subtraction algorithm with the primarily selected weight and the 2.5-times larger weight, we added intermediate weights.

In essence, the Mean-Subtraction algorithm subtracts a baseline value for each OCTA A-scan. The subtracted baseline is different for each OCTA A-scan, so the method is similar to the window setting. In some parts of the corrected OCTA image, the signal is missing because the original OCTA was lower than the subtracted value. When the weight is below the optimal value, the tail artifact is less effectively corrected—seen when comparing the first and the second row. When these images are compared (the first row with the *w*_2_ = 3 that is considered as an optimal and the second row with the *w*_2_ = 1.5), the differences appear to be small. Therefore, we consider that the range of optimal weight values is broad and manual adjustment is sufficient. If the weight is too high (third row in Figure 6), almost the whole image disappears, which is easy to notice. Due to this substantial difference, the correction with intermediate correction weight values is presented in Figure 7. As we see, the tail artifact is disappearing when the correction weight is increasing, and also the amount of vasculature visible on en face image is reduced.

The Step-down Exponential filtering method carries out a filtering along the OCTA A-scan direction. It reduces the OCTA tail artifacts, but the reduction is not complete. For any weight value, the image value stays above zero, which is expected from Equation (8). The difference is that all three images are hardly noticeable, so we consider that the range of optimal weight values is broad and manual adjustment is sufficient.

**Figure 9:**
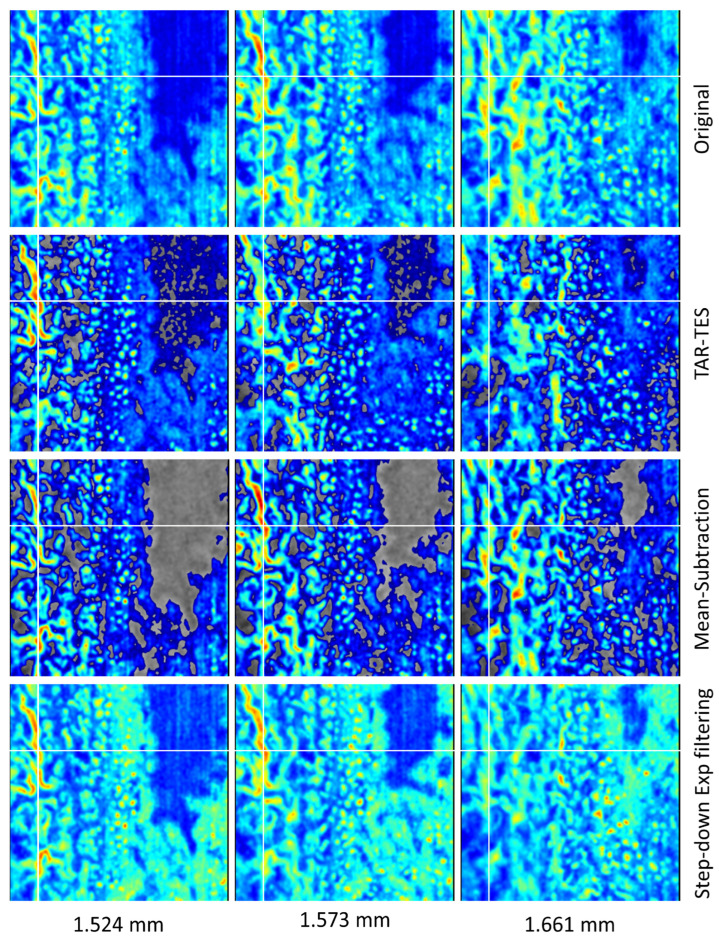
Original and corrected en face OCTA images at various depths. A correction was carried out with the TAR-TES method with *w*_1_ = 0.02, the Mean-Subtraction algorithm (MSA) with *w*_2_ = 3 and the Step-down Exponential filtering method (SDEF) with *w*_3_ = 0.02.

Additional insights into the TAR-TES algorithm performance, as compared to the Mean-Subtraction algorithm and the Step-down Exponential filtering method, can be gathered from Figure 9, which compares en face images at various depths. We can see that the vessel is clearly visible at depth of 1.524 mm on all images. Figure 4, Figure 5, Figure 6 and Figure 7 show en face image at depth of 1.540 mm, and we can see the selected vessel on all images very well. Going to a depth of 1.573 mm, we can see the differences between the correction algorithms; TAR-TES corrected OCTA already has relatively low signal for the vessel, whereas the original OCTA, the Mean-Subtraction algorithm and the Step-down Exponential filtering method have increased signal in comparison to the depth of 1.524 mm. At the depth of 1.661 mm, the vessel on the TAR-TES corrected OCTA has already vanished, whereas the original OCTA, the Mean-Subtraction algorithm and the Step-down Exponential filtering method still have clearly visible vessel.

**Figure 10:**
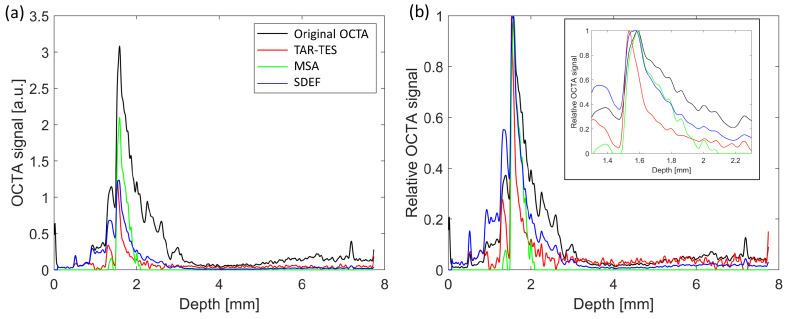
The A-scan plot of original and corrected OCTA—original values (**a**) and scaled values to the maximum (**b**). A correction was carried out using the TAR-TES method with *w*_1_ = 0.02, the Mean-Subtraction algorithm (MSA) with *w*_2_ = 3 and the Step-down Exponential filtering method (SDEF) with *w*_3_ = 0.02.

Another comparison of different OCTA correction methods is presented in Figure 10, which provides A-scan profiles of the original and corrected OCTA images for the point defined by the cross-center point in Figure 4, Figure 5, Figure 6, Figure 7, Figure 8 and Figure 9. As we see, all correction methods decrease the OCTA signal and change the shape of the depth profile. The tail artifact is seen as the slowly falling curve after the peak, while the correct shape should be a symmetric peak, i.e., the profile of the vessel (Figure 3). When the signal is normalized to its maximal value, we can compare the peaks and tail artifacts for different tail-correction algorithms. The Mean-Subtraction algorithm basically cuts the lower part of the curve while the shape of the upper part of the curve remains unchanged. The Step-down Exponential filtering method changes the shape of the curve. It reduces the tail artifact, but less than the other two methods. The proposed TAR-TES method is the most effective in reducing the tail artifact, which can be seen from the depth profile. For example, the OCTA signal at 0.2 mm beneath the peak falls to 20% of its maximal value in the case of the TAR-TES method, whereas the Mean-Subtraction and the Step-down Exponential filtering method reduce the OCTA signal to about 40% of its maximal value at this point. The uncorrected OCTA signal is at 60% of its maximal value. Interestingly, the TAR-TES method also moves the peak forward for about 0.05 mm. It should be noted that the diameter of the vessel is about 0.1 mm.

## 4. Discussion

The presented manuscript introduces the TAR-TES algorithm for correcting tail artifacts in Optical Coherence Tomography Angiography (OCTA) images and evaluates its performance compared to alternative correction methods. The results demonstrate the effectiveness of the TAR-TES algorithm in addressing tail artifacts and improving the quality of OCTA images.

The TAR-TES algorithm, with an appropriately adjusted weight constant *w*_1_, showed promising results in artifact removal. Generally, the weight constant *w*_1_ determines the relation between the reflectivity of the tissue, light transmittance to that point and OCT signal. This relation also implicitly includes the efficiency of the detection system. Altogether, it would be very difficult to determine the weight constant *w*_1_ from the basic principles. It remains the free parameter of the correction algorithm, which should be adjusted for the maximal efficiency of the algorithm. When adjusted properly, the TAR-TES algorithm strikingly reduced the tail artifacts while preserving the vasculature image’s integrity. The balance achieved in artifact removal is essential to ensure that the corrected OCTA images remain true to the underlying vasculature morphology.

The adjustment of weight constant *w*_1_ can be viewed as a regularization problem, where the appropriate regularization aggressiveness is necessary to achieve optimal results. An investigation into different weight constants shed light on the significance of parameter selection in the TAR-TES algorithm. A weight constant of *w*_1_ = 0.01 displayed partial tail artifact removal, suggesting that a lower weight is insufficient for comprehensive correction. On the other hand, *w*_1_ = 0.05 exhibited complete tail artifact removal but led to signal loss and alterations in vessel shape. This underscores the importance of fine-tuning the weight constant based on the specific characteristics of OCTA images and the desired level of artifact reduction. It is essential to note that the parameters we used are not the only viable choices and could be optimized in different ways. Additionally, different samples may require different parameter settings. Different scattering properties imply different magnitude of the transmission to deeper layers and different magnitude of reflected light, which implies that different parameter *w_1_* is optimal for each particular setting.

Comparative analysis with two alternative correction methods, namely the Mean-Subtraction algorithm and the Step-down Exponential filtering method, highlighted the advantages of the TAR-TES. The Mean-Subtraction algorithm, which subtracts a varying baseline value for each A-scan, reduces the OCTA signal after the vessels only because the baseline value is subtracted from the signal. The Step-down Exponential filtering method changes the shape of the A-scan profile of the OCTA signal. However, it did not completely eliminate tail artifacts. In contrast, the TAR-TES algorithm almost completely eliminated tail artifacts. On the other hand, the selected alternative methods may be less sensitive to the selection of the weight constant *w*_2_ and *w*_3_, with the exception of excessive correction weight in the case of the Mean-Subtraction algorithm that is easy to recognize. Due to this drastic reduction in the amount of vasculature in case of excessively aggressive correction with the Mean-Subtraction algorithm, we presented intermediate weights for the correction with this algorithm. In this case, the amount of vasculature is gradually decreasing, while the tail artifacts are also reduced. If we pick the specific vessel that we want to visualize, we can tune the Mean-Subtraction algorithm for this specific vessel so that only its tail artifact is removed while the vessel persists. However, such a procedure is almost the same as window setting in visualization. Therefore, its usefulness for the visualization is limited.

The A-scan profile analysis further clarifies how the TAR-TES algorithm reduces tail artifacts and enhances the depth-profile shape. The plot of the absolute signal values (Figure 10) shows that each correction method considerably reduces the OCTA signal strength. By normalizing the signal to its maximum value, it became evident that the TAR-TES algorithm is most successful in transforming the original OCTA signal into the expected symmetric peak.

The TAR-TES algorithm, while promising in addressing tail artifacts in Optical Coherence Tomography Angiography (OCTA) images, has several limitations. Its performance is sensitive to the selection of the weight constant *w*_1_, which can vary across datasets and imaging conditions. A high weight constant can lead to significant OCTA signal loss, potentially resulting in missing vasculature information. The algorithm may also alter the shape of vessels, which could impact the accuracy of quantitative vascular analysis. The performance of the algorithm can vary across different OCTA datasets due to the specific imaging conditions, patient populations, and imaging equipment used.

Depending on the clinical situation, motion artifacts can be a significant problem of the speckle-variance OCTA. Motion during the consecutive OCT scans also produce speckle patterns, similar as the blood flow, and therefore false flow image. One option to reduce this false flow signal is to perform complex-signal-based OCTA [7] or high-filtering of complex-signal-based OCTA [24]. Those techniques still suffer from the tail artifacts and the physics basis is similar as in the case of speckle-variance OCTA. Therefore, the ideas behind the TAR-TES algorithm may also be used for the tail artifact correction in these angiographic images, while the exact formulation may not be exactly as presented in this manuscript.

Possible future improvement in the TAR-TES algorithm for using it in the speckle-variance OCTA lies in the optimization of the parameter *w*_1_. An obvious improvement is the optimization of the algorithm on a broader range of datasets. Another possible direction is through the integration of artificial intelligence. The algorithm could be trained on simulated data or data obtained from phantom studies, enabling it to adapt and learn optimal parameter settings for the weight constant *w*_1_, tailored to specific imaging conditions and datasets. The incorporation of artificial intelligence would not only enhance the algorithm’s robustness and applicability across diverse scenarios but also make it more efficient and user-friendly. The third direction of future work is a modification of the algorithm by substituting the weight constant *w*_1_ with the function of depth. This improvement would correct the simplification that we do when going from Equation (4) to Equation (5), where we assume that the transmission factor *T_n_* is equal to 1.

While these incremental improvements in the TAR-TES algorithm could provide superb results, compared to existing algorithms, the idea behind the TAR-TES algorithm (especially the physical model behind) may also serve as a ground for new, better correction algorithms. The problem of the TAR-TES and Mean Subtraction algorithm is that they can yield non-physical negative amplitudes, which are subsequently set to zero (hence grey areas for those two algorithms in Figure 5 and Figure 6). Interestingly, the Step-down Exponential filtering does not suffer from this problem, which is obvious from Equation (8)—correction is carried out using an *always-positive* scaling factor instead of subtraction. If the correction is small, the correction for Step-down Exponential filtering can be derived as

(9) $$A_{n}^{corr}=A_{n}\times e^{-w_{3}\times \sum_{i=1}^{n-1}A_{i}^{corr}}\approx A_{n}\left(1-w_{3}\times \sum_{i=1}^{n-1}A_{i}^{corr}\right)$$

While this derivation is not valid for our case as the argument in the exponent for the location of vessels is close to 1, the form of Equation (8) is very similar to the TAR-TES equation (Equation (6)). One difference is the summation of corrections for shallower layers in squares and the other difference is scaling the correction with structural image *S_n_*. The latter is not a new idea as it is an essential part in the projection-resolved correction method [21].

The TAR-TES algorithm has the potential for widespread adoption and great impact on future clinical research and diagnostic applications of the OCTA. It is poised to enable true 3D vascular imaging by eliminating tail artifacts in OCTA images. This would enable true 3D reconstruction of vasculature topology and enhance the detection of deep-seated vessels beneath shallower vessels, providing a more accurate and comprehensive representation of complex vascular networks. This advancement holds significant promise in fields such as dermatology, ophthalmology, or oncology.

## 5. Conclusions

In conclusion, the TAR-TES algorithm emerges as a robust and valuable tool for correcting tail artifacts in OCTA images. Unlike the alternative correction methods, this algorithm is physics based. It provides enhanced image quality without sacrificing the accuracy of vessel morphology representation. This study underscores the importance of parameter optimization in the application of the TAR-TES algorithm and highlights its potential to improve the reliability of OCTA imaging in various clinical and research settings. Further research and validation across diverse datasets are warranted to fully harness the algorithm’s capabilities and ensure its broader applicability in the field of OCTA imaging.

## Data Availability

The data that support the findings of this study are available upon reasonable request from the authors.
